# Phytochemical, anti-inflammatory and analgesic properties of stembark extract and fractions of *Lonchocarpus sericeus *Poir. (Papilionaceae) in albino mice

**Published:** 2020

**Authors:** Uwemedimo Emmanuel, Johnbull Onyekachi, Okenwa Uchenna

**Affiliations:** 1 *Department of Chemistry, Faculty of Science, University of Uyo, P.M.B. 1017, Uyo, Akwa Ibom State, Nigeria.*; 2 *Department of Chemistry, College of Physical and Applied Sciences, Michael Okpara University of Agriculture, Umudike, P.M.B. 7267, Umuahia, Abia State, Nigeria.*

**Keywords:** Analgesia, Inflammation, Lonchocarpus sericeus, Phytochemicals

## Abstract

**Objective:**

*Lonchocarpus sericeus* stembark decoction has been extensively employed in folkloric medicine in many parts of Nigeria as a remedy for pain as well as inflammation. The plant was studied for its anti-inflammatory as well as analgesic potency using standard biological models.

**Materials and Methods:**

The stembark of *L. sericeus *was evaluated for anti-inflammatory properties using egg albumin and xylene-induced oedema models. The pain-relieving property was evaluated using acetic acid-induced writhing and thermally-induced pain models. Median lethal dose determination (intraperitoneal LD_50_), quantification of some phytochemicals as well as phytochemical screening were also performed.

**Results:**

The LD_50_ of stembark extract of *L. sericeus *was found to be 3,100 mg/kg (i. p). The crude extract and fractions (310-930 mg/kg) effectively reduced oedema caused by egg albumin and xylene and exhibited high analgesic properties in inhibiting pain induced by acetic acid and heat. These reductions were dose-dependent and statistically significant (p<0.05-0.001) when compared to distilled water and similar to prototype drugs employed. Quantitative determinations of some bio-active constituents of the plant showed a higher flavonoid content (0.52±0.02 mg/100 g) compared to alkaloids (0.36±0.02 mg/100 g) and flavonoids (0.49±0.03 mg/100 g). Phytochemical screening of the stembark showed the presence of alkaloids, cardiac glycosides, flavonoids terpenes, tannins and saponins.

**Conclusion:**

These results imply that the stembark extract of *L. sericeus * possesses anti-inflammatory and analgesic potency and these data validate its wide use in folkloric medicine for inflammation and pain management.

## Introduction

*Lonchocarpus sericeus* Poir. (Papilionaceae) is a leguminous plant which is known as Senegal lilac or Cube root. It is recognized as a dry deciduous tree with ability to grow from 10 to 16 meters high. It has flowers with dense hanging racemes of purple flowers which is perfect for display purposes. The flowers possess a characteristic smell which is similar to vanilla. It is commonly planted in villages as a shade tree and in gardens. The wood is clear yellow, sometimes marbled, with heartwood and olive-green (Kojs et al., 2004 ▶ and Adewuyi et al., 2012 ▶). 

In Nigeria, leaves are employed for broad-spectrum healing while the bark is exploited for management of body pains, arthritis, rheumatism, cutaneous and subcutaneous parasitic infection, malnutrition, debility, paralysis and convulsions. It is also used as fish-poisons and laxatives. The roots are used for treatment of leprosy. The fruit and seeds are used as insect repellants and arachnicides (Burkill, 1985 ▶).

There are scanty reports on the stembark of the plant. The available reports include: isolation of a pentacyclic triterpenoid lupeol from the stembark of *L. sericeus *(Abdullahi et al., 2013 ▶) as well as the anticonvulsant activity of methanol extract of the stem bark of *L. sericeus *(Musa et al., 2006 ▶). However, there is no report on the anti-inflammatory and analgesic activities of the stembark of *L. sericeus *despite its wide usage in ethnobotany, hence, the present study was carried out to validate this claim.

## Materials and Methods

**Plant materials**

The fresh stembarks of *L. sericeus* were sourced from a forest edge in Ikono Local Government Area of Akwa Ibom State, Nigeria and identified and authenticated by a botanist Mr. Ndukwe Ibe, from the Department of Forestry, Michael Okpara University of Agriculture, Umudike, Nigeria. The plant sample was further confirmed at the Pharmacognosy and Natural Medicine Department of the University of Uyo, Nigeria, where a voucher specimen (UUH 62/19) of the plant was deposited in the Herbarium

**Extraction and partitioning**

The plant part (stembarks) was washed and shade-dried for two weeks. The dried stembarks were further chopped into small pieces. The chopped stembarks (2.0 kg) was macerated in 97% methanol for 72 hr to give the crude methanol extract. The liquid filtrate was concentrated and evaporated to dryness *in* *vacuo* at 40°C using a rotary evaporator. The dried crude extract was deposited in a refrigerator at 4°C prior to use for the proposed experiment. 

The methanol extract of *L. sericeus *stembark (100 g) was dispersed in 300 ml of distilled water and partitioned into hexane and dichloromethane using a separating funnel. All fractions were subsequently concentrated under reduced pressure in a rotary evaporator (WG-EV311-V, Wilmad-LabGlass, USA) at 40°C until they became completely dry. The hexane fraction (17.0 g) and Dichloromethane fraction (20.0 g) were kept in a sealed container and preserved in a refrigerator maintained at 4°C until analysis. 

**Phytochemical screening**

Phytochemical screening of the crude extract was performed using conventional analytical procedures (Trease and Evans, 1996) to assess the presence of chemical components such as alkaloids, anthraquinones, cardiac glycosides, flavonoids, tannins, terpenes, saponins and phlobatannins; also, quantitative determination of alkaloids was done by the method described by Harborne (1973) ▶ while saponin was assessed by the method used by Obadoni and Ochuko (2001) ▶ and flavonoids by the method of Bohm and Kocipai-Abyazan (1994) ▶. 

**Animals**

Adult albino mice (weight: 20-30 g) of both sexes were employed in these experiments. They were all sourced from the Animal House of the Department Pharmacology and Toxicology, University of Uyo, Uyo. They were placed in well ventilated cages in groups (n=5) for the experiments. The animals were sustained on standard livestock pellets (Guinea Feed Nigeria Ltd.) and water *ad libitum* and maintained under standard laboratory conditions (12 hr light and dark cycles). The animals were taken out of the animal house and acclimatized to the laboratory environment for about 2 hr prior to commencement of pharmacological studies. The care and handling of these animals were carried out in strict compliance with the current guidelines of the International Association for the study of pain, for the use of animals in pain research (Zimmermann, 1986 ▶).

**Determination of median lethal dose (LD**_50_ **)**

The median lethal dose (LD_50_) of the plant extract administered intraperitoneally (i.p.), was assessed in albino mice by using the method of Lorke (1983) ▶. Twenty-one albino mice were selected and divided into groups of three mice per group. Prior to the experiment, animals underwent 24 hr starvation but they had access to water. The extracts were administered in a dose range of 500-3,500mg/kg body weight of animals. The animals were observed for physical signs of toxicity such as writhing, decreased motor activity, decreased body/limb tone, decreased respiration, and death. The number of deaths in each group within 24 hr, was recorded. The LD_50_ was calculated as the geometrical means of the maximum dose producing 0% mortality (D_0%_) and the minimum dose producing 100% (D_100%_) mortality. That is,

${\mathrm{LD}}_{50}=\sqrt[{2}]{D_{0}\%\times D_{100}}\%$

**Evaluation of anti-inflammatory activity**

 The following experimental models were used to study the anti-inflammatory properties of the methanol stembark extract as well as the fractions.

**Egg albumin-induced inflammation**

Adult albino mice of both sexes (20-30 g) were divided into 10 groups of five animals each for the extract (methanol) and fractions (hexane, DCM and aqueous). The animals were used for the experiment after 24 hr fast and were only deprived of water during the experiment. Inflammation of the hind paw was induced by injecting 0.1 ml of fresh egg albumin (Phlogistic agent) subcutaneously (s.c.) into the sub-plantar surface of the right hind paw (Akah and Nwambie, 1994 ▶). The control group (group 1) was pre-treated with 10 ml/kg distilled water and the reference group (group 8) received 100 mg/kg acetyl salicylic acid i.p.. The test groups (groups 2-7) of mice were pre-treated with 310, 620 and 930 mg/kg of the extract as well as 160 mg/kg of n-hexane, DCM and aqueous fractions (i.p.), respectively. The linear circumference of the injected paw was measured before administering the fresh egg albumin (C_0_) and every 30 min thereafter for 5 hr (C_t_) using digital veneer caliper (Tianheng Tools Co. Ltd, China). The difference between C_1 _and C_0_ within a given time, represented the degree of inflammation (Hess and Milonig, 1972 ▶). Results were calculated according to the standard formula:

$\mathrm{Percentage Inhibition}=\frac{(C_{t}-C_{0}{)}_{\mathrm{control}}-(C_{t}-C_{0}{)}_{\mathrm{treated}}}{(C_{t}-C_{0}{)}_{\mathrm{control}}}\times 100$

Where:

C_t_=paw circumference at time t

C_0_=paw circumference before fresh egg albumin was injected 

C_t_-C_0_=oedema

**Xylene-induced ear edema**

Inflammation was induced in mice by topical administration of two drops of xylene at the inner surface of the right ear. The xylene was left to act for 15 min. *L. sericeus* stem extract (310, 620 and 930 mg/kg, i.p.), dexamethasone (4 mg/kg, orally) and distilled water (0.2 ml/kg, orally) were administered to various groups of mice 30 min before the induction of inflammation. The animals were sacrificed under light anesthesia and the left ears were cut. The difference between the ear weights was taken as the edema induced by the xylene (Mbagwu et al., 2007 ▶; Okokon and Nwafor, 2010 ▶; Tjolsen et al., 1992 ▶).

**Evaluation of analgesic activity of** ***L. sericeus***

*Acetic acid-induced writhing in mice*

Writhing (abdominal constrictions consisting of the contraction of abdominal muscles together with the stretching of hind limbs), resulting from i.p. injection of 3% acetic acid, was induced according to the procedure earlier described by Santos et al. (1994) ▶, Correa et al. (1996) ▶ and Nwafor et al. (2010) ▶. The animals were divided into five groups of five mice per group. Group 1 served as negative control and received 10 ml/kg of normal saline, while groups 2, 3 and 4 were pre-treated with 310, 620 and 930 mg/kg doses of *L. sericeus* i.p., and group 5 received 100 mg/kg of acetyl salicylic acid. After 30 min, 0.2 ml of 2% acetic acid was administered i.p.. The number of writhing movements was counted for 30 min. Antinociception (analgesia) was expressed as the reduction of the number of abdominal constrictions in control animals and mice pretreated with extracts.

**Thermally-induced pain in mice**

The effect of extract on hot plate-induced pain was studied in adult mice. The hot plate was used to measure the response latencies according to the method earlier put forward by Vaz et al. (1996) ▶ and Okokon and Nwafor (2010) ▶. In this experiment, the hot plate was maintained at 45±1℃. The animals were placed into a glass beaker of 50 cm diameter on a heated surface, and the time(s) between placement and shaking or licking of the paws or jumping was recorded as the index of response latency. An automatic 40 sec cut-off was used to prevent tissue damage. The animals were randomly divided into five groups of six mice each and fasted for 24 hr but allowed access to water. Group 1 animal served as negative control and was given 10 ml/kg of distilled water. Groups 2, 3 and 4 were pretreated i.p. with 310, 620 and 930 mg/kg doses of *LS* extract, respectively, while group 5 was given 100 mg/kg of acetyl salicylic acid i.p., 30 min before the placement on the hot plate.

**Statistical analysis**

Multiple comparisons of mean+SEM were carried out by one-way analysis of variance (ANOVA) for thermally-induced pain model and xylene-induced ear oedema model while two-way ANOVA was used for egg-albumin-induced right hind paw oedema and acetic acid-induced writhing models followed by Bonferroni and Tukey’s multiple comparisons tests using Graph pad prism software package 8.0 operated on Windows 10 platform. Any probability level of less than 5% was considered significant.

## Results

**Phytochemical screening**

The result of the phytochemical screening of the methanol stembark extract of *L. sericeus *is presented in Table 1, while that of the quantitative determination of som

**Anti-inflammatory studies**

**Egg albumin-induced inflammation**

The effect of methanol stembark extract of *L. sericeus*, n-hexane, dichloromethane and aqueous fractions on fresh egg albumin-induced inflammation on mice hind paw oedema, is presented in Table 3. 

**Table 1:** Result of phytochemical screening of methanol stembark extract of *L. sericeus*

**Phytochemical**	**Tests/Reagents**	**Result **
Alkaloid Anthraquinones Cardiac glycosides Flavonoids Terpenes/steroids Phlobatannins Saponins Tannins	Dragendroff’s Mayer’s Benzene/ammonia solution Liebermann’s Keller-Killiani’s Magnesium metal, HCl Chloroform, H_2_SO_4_ acid HCl acid solution Frothing test Emulsion test Ferric chloride solution	Present Present Absent Present Present Present Present Present Absent Present Present Present

**Table 2:** Quantitative determination of some bioactive compounds of stembark extract *Lonchocarpus sericeus*

**Plant constituents**	**Alkaloids**	**Flavonoids **	**Saponins**
Concentration (mg/100 g)	0.36±0.02	0.52±0.02	0.49±0.03

Results are given as mean±standard error of mean. Each determination was done in triplicate.

**Xylene-induced oedema**

The effect of methanol stembark extract of *L. sericeus*, n-hexane, dichloromethane (DCM) and aqueous fractions on xylene-induced topical oedema in mice right ear, is presented in Table 4.

**Table 3:** Effect of methanol extract and fractions of *L. sericeus *stembark on Egg-albumin-induced right hind paw oedema in mice

**Treatment** **(mg/kg)**	**Time (hours)**					
	**0**	**1**	**2**	**3**	**4**	**5**
Control Extract 310 Extract 620 Extract 930 n-hexane 620 DCM 620 Aqueous 620 ASA 100	2.336±0.015 2.254±0.022 2.342±0.052 2.410±0.051 2.406±0.066 2.344±0.061 2.304±0.066 2.270±0.043	0.724±0.025 0.668±0.051 0.616±0.043 0.514±0.021^+++^ 0.580±0.070^+^ 0.694±0.045 0.606±0.038 0.510±0.030^+++^	0.688±0.028 0.552±0.046 0.424±0.025^+++^ 0.400±0.024^+++^ 0.362±0.031^+++^ 0.412±0.069^+++^ 0.466±0.032^+++^ 0.460±0.029^+++^	0.626±0.074 0.396±0.024^+++^ 0.358±0.025^+++^ 0.362±0.016^+++^ 0.300±0.034^+++^ 0.374±0.077^+++^ 0.386±0.035^+++^ 0.268±0.004^+++^	0.538±0.037 0.338±0.030^++^ 0.236±0.024^+++^ 0.214±0.022^+++^ 0.196±0.030^+++^ 0.268±0.032^+++^ 0.308±0.038^+++^ 0.162±0.019^+++^	0.484±0.046 0.210±0.015^+++^ 0.182±0.035^+++^ 0.164±0.027^+++^ 0.122±0.014^+++^ 0.164±0.045^+++^ 0.256±0.034^+++^ 0.078±0.017^+++^

Values are expressed as mean±SEM; (n=5);

Significance relative to control; ^+^p<0.05, ^++^p<0.01, and ^+++^p<0.001.

ASA=Acetyl salicylic acid.

**Table 4:** Effect of methanol stembark extract and fractions of *L. sericeus *on xylene-induced ear oedema in mice

**Treatment (mg/kg)**	**Weight difference (g)**
Distilled Water (10 ml/kg) Extract 310 Extract 620 Extract 930 n-hexane 620 DCM 620 Aqueous 620 Dexamethasone 4 mg/kg	0.02370±0.0008 0.0115±0.0020^+++^ 0.0104±0.0024^+++^ 0.0069±0.0009^+++^ 0.0138±0.0019^++^ 0.0060±0.0016^+++^ 0.0144±0.0016^++^ 0.0048±0.0003^+++^

Values are expressed as mean±SEM; (n=5)

Significance relative to control; ^+^p<0.05, ^++^p<0.01, and ^+++^p<0.001

**Analgesic study**

**Acetic acid writhing**

The effect of methanol stembark extract of *L. sericeus*, n-hexane, dichloromethane and aqueous fractions on acetic acid-induced writhing in mice, is presented in Table 5.

**Thermally-induced pain**

The result of the methanol stembark extract, aqueous, n-hexane and dichloromethane fractions is presented in Table 6.

## Discussion

**Phytochemical analyses**

The yield of crude methanol extract was 6.00%. The result of phytochemical analyses of the methanol stembark extract of *L. sericeus*, as presented in Table 1, revealed that the extract contained alkaloids, saponins, cardiac glycosides, flavonoids terpenes and tannins. Anthraquinones and phlobatannins were not found. Quantitative determinations of some bioactive constituents (Table 2), showed a higher flavonoid content compared to alkaloids and saponins.

The presence of these phytochemicals in the stembark of *L. sericeus* makes it advantageous to the consumer because these compounds have demonstrated potent medicinal activities, including analgesic, anticancer, bactericidal, wound healing, hepatoprotective, anti-inflammatory, and antioxidant properties (Pandey et al., 2012 ▶; Bribi et al., 2017 ▶; Mishra et al., 2011 ▶; Weina et al., 2017 ▶; Zhu et al., 2012 ▶; Boakye et al., 2018 ▶).

**Table 5:** Effect of stembark extract and fractions of *L. sericeus *on acetic acid –induced writhing in mice

**Treatment** **(mg/kg)**	** Time (minutes)**					
	**5**	**10**	**15**	**20**	**25**	**30**
Control Extract 310 Extract 620 Extract 930 n-hexane 620 DCM 620 Aqueous 620 ASA 100	8.200±3.02 5.00±2.86 4.40±1.66 3.40±1.28 3.60±0.40 0.40±0.24 2.40±0.74 0.40±0.24	18.20±6.53 7.60±1.60^+^ 8.20±1.59^+^ 6.40±1.66^++^ 4.20±0.37^+++^ 2.60±0.60^+++^ 4.40±0.74^+++^ 0.80±0.37^+++^	20.20±3.55 9.20±2.45^++^ 7.80±3.10^++^ 7.00±1.26^+++^ 4.40±0.40^+++^ 3.20±0.86^+++^ 7.00±1.64^+++^ 3.20±080^+++^	25.20±2.95 10.40±2.46^+++^ 7.00±3.05^+++^ 6.60±1.12^+++^ 5.20±0.97^+++^ 4.00±0.54^+++^ 7.40±0.81^+++^ 4.00±0.44^+++^	23.40±4.91 10.40±4.15^+++^ 10.80±3.94^++^ 4.20±2.70^+++^ 4.40±0.24^+++^ 1.60±0.60^+++^ 13.00±1.26^+^ 3.60±1.03^+++^	30.40±3.98 7.60±2.40^+++^ 9.20±4.24^+++^ 3.20±1.82^+++^ 3.80±0.66^+++^ 2.40±0.24^+++^ 9.20±0.58^+++^ 2.80±0.37^+++^

Values are expressed as mean±SEM; (n=5)

Significance relative to control; ^+^P <0.05, ^++^P<0.01, ^+++^P <0.001

ASA=Acetyl salicylic acid

**Table 6:** Effect of methanol extract and fractions of *L. sericeus *stembark on thermally-induced pain in mice

**Treatment (mg/kg)**	**Reaction time (seconds)**
Distilled water (10 ml/kg)	7.922±0.590
Extract 310 Extract 620 Extract 930 *n*-hexane 620 DCM 620 Aqueous 620 ASA 100	18.980±1.558 21.020±1.429^+^ 21.900±1.717^+^ 25.490±4.411^++^ 22.480±2.734^+^ 18.140±2.703 40.000±0.000^+++^

Values are expressed as mean±SEM; (n=5)

Significance relative to control; ^+^p<0.05, ^++^p<0.01, and ^+++^p<0.001

ASA=Acetyl salicylic acid

Alkaloids are recognized as one of the classes of therapeutically active plant substances. Pure, isolated, and synthetic derivatives are quite useful as basic medicinal agents because of their analgesic, anti-nociceptive, antioxidant and intestinal anti-inflammatory activities (Bribi et al., 2013 ▶; Bribi et al., 2015 ▶; Bribi et al., 2017 ▶). 

Saponins have been credited with a number of pharmacological properties. However, the important ones include permeabilizing of the cell membrane (Hostettmann and Marston, 1995 ▶), lowering serum cholesterol levels (Francis et al., 2002 ▶), stimulation of luteinizing hormone release leading to abortifacient properties (Francis et al., 2002 ▶), immunomodulatory potential via cytokine interplay (Sun et al., 2009 ▶), cytostatic and cytotoxic effects on malignant tumor cells (Bachran et al., 2008 ▶), adjuvant properties for vaccines as immune-stimulatory complexes (Sjolander et al., 1998 ▶), and synergistic enhancement of the toxicity of immunotoxins (Heisler et al., 2005 ▶).

Flavonoids are quite renowned for their anti-oxidant, hepatoprotective and anti-cancer potentials (Kumar and Pandey, 2013 ▶. Flavonoids are also known for their anti-inflammatory and allergic effect coupled with their gastric mucus production (Pan et al., 2010 ▶). Flavonoids possess some antibacterial and antifungal properties (Akpan et al., 2012 ▶; Mishra et al., 2013 ▶). It is safe to suggest that these bioactive compounds found in the plant stembark could be responsible for the various pharmacological activities of the plant and corroborate its widespread use in traditional medicine.

The acute toxicity test indicated that the administration of the crude methanol stembark extract at a dose of 4000 mg/kg resulted in 100% mortality of the mice in the various groups tested, while no mortality was observed in the group administered with 2,000, 2,500 and 2,750 mg/kg of the sample. The median lethal dose (LD_50_) of the stembark extract of *L. sericeus* was calculated as 3,100 mg/kg and the various doses of the extracts used for the study were calculated as 310, 620 and 930 mg/kg of the lethal dose which represented the low, median and high doses of the extracts. 

The LD_50_ (i.p.) value of 3,100 mg/kg for the methanol extract obtained in mice, indicated high safety profile. The LD_50_ of a sample is the dose that kills 50% of the animals in each group. Before death, the animals exhibited decreased locomotion, tremor, uncoordinated body movement and convulsion. In general, the smaller the LD_50_ value, the more toxic the sample is and the larger the LD_50 _value, the lower the toxicity with respect to the same route of administration. It therefore means that a large quantity of the material will be required to bring about a toxic response. 

**Anti-inflammatory studies:**

The anti-inflammatory effects of the methanol extract and partitioned fractions of the stembark of *L. sericeus *on egg albumin-induced oedema in mice hind paw are shown in Table 3. These results indicate that the methanol extract as well as the hexane and DCM fractions demonstrated considerable anti-inflammatory potential which were comparable to the standard, acetyl salicylic acid (ASA). Specifically, the high dose (930 mg/kg) of the methanol extract demonstrated the same level of potency like the standard drug after one hour of pretreatment and showed a similar trend throughout the period of study. 

The result of the study which indicated the ability of the extracts of *L. sericeus *to suppress paw diameter induced by egg albumin, a phlogistic agent in a manner similar to ASA, a cyclo-oxygenase inhibitor, suggests that the extract has systemic potential in the inhibition of oedema. The result of phytochemical screening of the extract did reveal the presence of many secondary metabolites which are implicated in intrinsic anti-inflammatory properties, hence the ability of these extracts in inhibiting paw oedema may not be unconnected with these compounds (Dahham et al., 2015 ▶; Chirumbolo, 2010 ▶).

The effect of methanol extract as well as the partitioned extracts of the stembark of *L. sericeus *on xylene-induced ear oedema in mice ear, as shown in Table 4, indicated that the extracts also inhibited (p<0.001) oedema caused by the topical administration of xylene at all dose regimes, when compared to distilled water in a way similar to the prototype drug, dexamethasone. 

In systemic anti-inflammation study, two mechanisms of inhibition of oedema are recognized. The first being the local release or formation of autacoids such as histamine, 5-hydroxytryptamine (5-HT) kinins, prostanoids while the second is the neurogenic stimulation of primary sensory neurons followed by the release of prostaglandins, a mediator of inflammation (Ajaghaku et al., 2013 ▶; Lembeck and Holzer, 1979 ▶). Just like ASA, the possible mechanism (s) of the extracts and isolated compounds of *L. sericeus *in inhibiting oedema by egg albumin, may in part, be due to their ability to block these inflammatory sequences.

The induction of oedema by xylene is linked to the release of phospholipase A_2_, therefore, the ability of the extract and fractions to inhibit oedema caused by xylene may be due to the blocking of the release of phospholipase A_2 _(Lin et al.,1992 ▶).

Lipophilic constituents are known to exhibit topical anti-inflammatory activity (Maria et al., 2007 ▶) by enhancing the permeability of the skin to anti-inflammatory drugs and even when in combination (Okoye et al., 2010 ▶). The ability of the *n*-hexane and dichloromethane fractions in inhibiting topical oedema, may also in part, be due to its lipophilic nature (Ajaghaku et al., 2013 ▶). 

**Analgesic study**

The effect of the methanol extract as well as the partitioned extracts of *L. sericeus *on acetic acid-induced writhing in mice, are presented in Table 5. The result revealed the ability of the extract and partitioned fractions to reduce acid-abdominal constrictions and stretching of mice hind limbs in a dose-dependent manner. These reductions were statistically (p<0.001) significant relative to distilled water but not as potent as ASA. 

The effect of methanol stembark extract and partitioned fractions of* L. sericeus *on thermally-induced pain in mice, is presented in Table 6. The extracts showed a dose-dependent analgesia against thermally-induced pain in mice. The fractions showed improved analgesic activity but not as potent as ASA. The inflammatory pain produced by acetic acid acts by causing capillary permeability and thermally induced pain is indicative of the extracts, fractions and isolated compounds behaving like narcotics. Thus, the ability of reducing pain may be related to their intrinsic anti-inflammatory and narcotic effects (Nwafor and Okwuasaba, 2003 ▶).

These results suggest that the stembark extract of *L. sericeus * possesses anti-inflammatory and analgesic activities and these data validate its use in ethnomedicine for management of inflammation and pains. The high LD_50_ (i.p.) confirms the very low toxicity level of the stembark decoction of *L. sericeus. *The administration (i.p.) of the stembark decoction of *L. sericeus *at these dose regimes is therefore not expected to produce harmful results.
